# OMICs approaches-assisted identification of macrophages-derived MIP-1γ as the therapeutic target of botanical products TNTL in diabetic retinopathy

**DOI:** 10.1186/s12964-019-0396-5

**Published:** 2019-07-22

**Authors:** Ning Wang, Cheng Zhang, Yu Xu, Sha Li, Hor-Yue Tan, Wen Xia, Yibin Feng

**Affiliations:** 1 School of Chinese Medicine, The University of Hong Kong, 1/F, 10 Sassoon Road, Pokfulam, Hong Kong, S.A.R. China; 2 Joint Research Center for National and Local Miao Drug, Anshun, Guizhou Province People’s Republic of China

**Keywords:** Tang-Ning-Tong-Luo, Diabetic retinopathy, Inflammation, Endothelial dysfunctions, MIP1γ/CCR1 axis, Retina macrophages

## Abstract

**Background:**

Inflammatory reaction in the dysfunction of retinal endotheliocytes has been considered to play a vital role in diabetic retinopathy (DR). Anti-inflammatory therapy so far gains poor outcome as DR treatment. This study aims to identify a novel therapeutic target of DR from the OMICs studies of a traditional anti-DR botanical products TNTL.

**Methods:**

Hyperglycemic mice were treated with TNTL. The anti-hyperglycemic effect of TNTL was validated to confirm the biological consistency of the herbal products from batches. Improvement of DR by TNTL was examined by various assays on the retina. Next-generation transcriptome sequencing and cytokine array was used to identify the therapeutic targets. In vitro study was performed to validate the target.

**Results:**

We observed that TNTL at its high doses possessed anti-hyperglycemic effect in murine type I diabetic model, while at its doses without reducing blood glucose, it suppressed DR incidence. TNTL restored the blood-retina barrier integrity, suppressed retinal neovascularization, and attenuated the retinal ganglion cell degeneration. Transcriptomic analysis on the retina tissue of hyperglycemic mice with or without TNTL revealed that the inflammatory retina microenvironment was significantly repressed. TNTL treatment suppressed pro-inflammatory macrophages in the retina, which resulted in the inactivation of endothelial cell migration, restoration of endothelial cell monolayer integrity, and prevention of leakage. Cytokine array analysis suggested that TNTL could significantly inhibit the secretion of MIP1γ from pro-inflammatory macrophages. Prevention of endothelial dysfunction by TNTL may be mediated by the inhibition of MIP1γ/CCR1 axis. More specifically, TNTL suppressed MIP1γ release from pro-inflammatory macrophages, which in turn inhibited the activation of CCR1-associated signaling pathways in endothelial cells.

**Conclusion:**

Our findings demonstrated that TNTL might be an alternative treatment to DR, and the primary source of potential drug candidates against DR targeting MIP1γ/CCR1 axis in the retinal microenvironment.

## Background

As one of the characteristic microvascular complications of diabetes mellitus (DM), diabetic retinopathy (DR) is the leading cause of vision loss in diabetic patients [1]. In particular, two studies on the incidence of DR in Chinese patients indicated that the prevalence of DR in Hong Kong accounts for 24.8 and 39.2% at baseline [2]. DR initially consists of an early non-proliferative stage but progresses into proliferative DR mainly associated with hyperglycemia and glucose dyscontrol [3]. Vascular endothelial growth factor (VEGF) plays a crucial role in mediating the progression of DR [4]. VEGF is produced in the retina with hypoxia-inducible factor-1α (HIF-1α) as the responsible transcriptional factor. Accumulation of VEGF in retina stimulates the generation of neovascularization as well as leakage of neighboring capillaries, which contributes to the blurred vision and eventually retinal damage [5]. Given its critical role in the pathological progression of DR, anti-VEGF treatment has been used to reduce neovascularization, though both panretinal photocoagulation and vitrectomy are yet the first-line and effective available therapy for DR [6]. Anti-VEGF treatment, majorly consisting of various antibodies to VEGF analogs, is useful but failed to be long-term economically affordable due to its considerable cost for DR patients. Moreover, chronic patients were found not well responsive to anti-VEGF treatment [3].

Inflammation has been considered as a central role player in the initiation and development of DR in DM patients. Of note, increasing clinical evidence has postulated the association between inflammation and clinical features of DR. Up-regulation of pro-inflammatory cytokines and chemokines, such as TNFα, IL6, IL1β, and MCP-1, were detected in plasma and vitreous samples of patients with DR [7]. Serum levels of pro-inflammatory TNFα (*p* = 0.013), CRP (*p* = 0.002) and VEGF (*p* = 0.003) were significantly higher in Type II DM patients with DR than those without DR [8]. A study within American Africans with type I DM revealed that serum pro-inflammatory factor TNFα was correlated with the prevalence of DR (*p* < 0.001), proliferative DR (*p* < 0.001) and the incidence of diabetic macular edema (DME) (*p* < 0.001) [9]. Meanwhile, pro-inflammatory TNFα, IL6, IL1β, IL8, and sIL2R levels significantly increased during the progression of non-proliferative DR towards proliferative DR [10]. The early inflammation-related events in DR involved the release of pro-inflammatory cytokines and adhesion of leukocytes to the retinal vasculature, lead to compromised vascular cells, tight junctions, and consequently, vascular leakage [11]. Increasing lines of evidence have revealed that inflammation in the retina was caused by hyperglycemia, the leading risk factor of DR in DM [12]. High glucose-induced activation of the resident and structurally-essential cells in the retina, is now known to be the facilitator of an inflammatory reaction during DR initiation [13]. Unfortunately, outcomes of trials of localized and systemic treatment using currently available anti-inflammatory agents remain controversial and discouraging. Developing novel anti-inflammatory treatments for DR is necessarily urgent.

In this study, we used multiple OMICs approaches to identify the novel therapeutic target in the treatment of diabetic retinopathy by a herbal product Tang-Ning-Tong-Luo (TNTL). TNTL has been clinically used as traditional Chinese Miao medicine for centuries to treat diabetic-like symptoms of indigenous people in the mountain area. We observed that high doses of TNTL might possess hypoglycemic effects, while at non-hypoglycemic doses, TNTL was able to suppress DR incidence and progression in type I diabetic murine model. Transcriptomic analysis was then performed to identify the global change of retinal gene expression after TNTL treatment. Gene ontology and pathway analysis revealed that TNTL primarily suppressed inflammation in the retina of diabetic mice. Histological analysis showed that TNTL reduced the presence of inflammatory macrophages in the retina, and resulted in inactivation of endothelial cells. Cytokine profile analysis suggested that suppression of MIP1γ release from macrophages by TNTL may contribute to its inhibitory effects on endothelial dysfunction. Our present study suggests that TNTL may be an alternative treatment for DR, and a potential lead for the discovery of novel anti-inflammatory agents that are suitable for DR treatment.

## Materials and methods

### Preparation of TNTL

TNTL is an ethnomedical formula commonly used in traditional Chinese Miao medicine by the indigenous people in mountainous areas of southwestern China. It was composed of Plantagins Herba (Cheqiancao in Chinese), Lonicerae Flos (Shanyinghua in Chinese), Agrimoniae Herba (Xianhecao in Chinese) and Trichosanthis Radix (Tianhuafen in Chinese). Production of TNTL was performed in GMP manufacturing in Bailing Pharmaceutical Co. (Guizhou, China).

### Cell line and cell culture

Retinal Endothelial Cells (RECs) were purchased from Sciencell. RECs were cultured in Endothelial Cell Medium (Sciencell, USA) with 5% fetal bovine serum, 1% endothelial cell growth supplement and 1% penicillin/streptomycin solution at 37 °C incubator with 5% CO_2_.

### Animal study

Protocols of animal study were reviewed and approved by the Committee on the Use of Live Animals in Teaching and Research of the University of Hong Kong. Streptozotocin (STZ)-induced hyperglycemic mice were utilized as type I diabetic-like model associated with retinopathy. The 10-week C57/BL/J male mice received 5 constitutive intraperitoneal injections of 50 mg/kg STZ in a citric buffer (pH 4.5). Five days after the last injection, 4 h-fasting blood glucose (FBG) was determined, and only FBG within 15.0 to 20.0 nmol/L were included. Mice only injecting citric buffer was used as normal control. Hyperglycemic mice were randomized into 5 groups: model, insulin treatment, and three TNTL treatment groups. For insulin group, mice received a daily injection of insulin (0.1 U/10 g b.w., i.p.). For TNTL treatment groups, mice received different doses of TNTL solution in water (0.9, 1.8, and 3.6 g/kg b.w./day) via gavage. Normal and model groups of mice received the same volume of water. Treatment lasted 4 weeks. Body weight and random blood glucose were tested weekly. At the end of this experiment, FBG and glucose tolerance tests were performed. To measure glucose tolerance, mice were fasted for 12 h and then received 2 g/kg b.w. glucose via intraperitoneal injection. The blood glucose level at 0, 30, 60, 90, and 120 min post-injection was determined and plotted. Evans blue assay was used as a direct indicator of retinal vascular leakage. Evans blue was intravenously injected to the mice and dye leakage onto the retina due to blood-retinal barrier breakdown was determined by dissecting the retina and extraction of Evans blue dye according to our previous publication [14]. The area under the curve of the plot was calculated. Serum was collected, and HbA1c was measured according to the manufacturer’s instruction.

### Histology and retinal vascular preparation

Eyeballs were collected from sacrificed mice. The retina was enucleated and placed on 4% paraformaldehyde overnight. The fixed retina was dehydrated and embedded. 4 μm paraffin section of the retina was stained with hematoxylin and eosin dye for histological measurement. For retinal vascular preparation, the fixed retina was digested with 3% of trypsin dissolved in 0.1 M Tris buffer (pH = 8.2), and the preserved vascular architecture was stained with hematoxylin-eosin dye and imaged under 400x magnification by a digital camera (CoolSNAP) linked to the microscope (LEICA). The number of endothelial cells/pericytes and acellular capillaries were quantified in each sample.

### Wholemounting

Survival RGCs were labeled by specific markers RBPMS and Tuj1 following a previous procedure with modifications [15, 16]. Briefly, after mice were anesthetized by intraperitoneal injection of pentobarbital (200 mg/kg), eyeballs were enucleated and immediately fixed in 4% paraformaldehyde (PFA) for 24 h. Afterwards, eyecups were taken out and post-fixed in 4% PFA for 6 h followed by making four cuts to lie flat the retina as whole-mount tissue. Retinas were slightly rinsing five times in PBS for 10 mins per wash and then incubated in 3% Triton X-100 / 2% DMSO mixture (Sigma-Aldrich, St. Louis, MO) in PBS for 3 days at 4 °C. Then, retinas were moved to blocking solution, including 10% goat serum and 3% Triton X-100 in PBS, at room temperature for 4 h. Afterwards, retinas were immunostained with the antibody βIII-tubulin (Tuj1) and RBPMS respectively (1:300, Abcam, USA). Sites that bound to primary antibody were visualized by incubating with Alexa Fluor 488 or 568-conjugated antibodies to corresponding IgG (1:500, Invitrogen, USA) for 6 h, respectively. Following the final rinsing (PBS for 2 times), retinas were whole flat-mounted on glass slides with RGC layer up. RBPMS/Tuj1-double positive cells in RGC layer are identified as survival RGCs. Image J was applied for counting RBPMS^**+**^ /Tuj1^**+**^ RGC cells from eight random fields taken from four angles of the retina (0°, 90°, 180°, and 270°), four at both 1 mm and 2 mm from the optic nerve head respectively. Each selected area was 0.076 mm^2^ (250 × 250 μm). All images were captured by a confocal laser microscope (200×, Carl Zeiss LSM 780, USA).

### Transcriptomic analysis and bioinformatics study

Total RNA was isolated from the retina tissue of hyperglycemic mice with or without TNTL treatment using RNeasy mini kit (Qiagen, Germany). The total RNA was then subject to library construction and transcriptomic analysis (Exiqon, Denmark). The original data of mRNA sequencing was then uploaded to NetworkAnalyst platform for further analysis (https://www.networkanalyst.ca) [17–20]. Differential analysis of the normalized gene expression was performed with DESeq2. Genes with a significant change in expression (adjusted *p*-value< 0.05, log2(fold change) > 1) were shortlisted for the generation of heatmap and volcano plot, as well as the analysis of protein-protein interaction network and gene set enrichment analysis (GSEA). Gene ontology (GO) and KEGG pathway analysis were performed on DAVID platform (https://david.ncifcrf.gov/home.jsp) with common genes of the three retrieved lists.

### Bone marrow-derived macrophages (BMDMs) preparation

BMDMs were prepared according to our previous publication [21]. In brief, cell suspension from femurs of C57/BL/J mice was collected with Ficoll method and induced for differentiation with murine recombinant macrophage-colony stimulating factor (M-CSF, 10 ng/mL) in RPMI1640 medium supplemented with 10% FBS. After 7-day of stimulation, BMDMs were induced into pro-inflammatory phenotype after addition of IFNγ (20 ng/mL) and lipopolysaccharide (LPS, 100 pg/mL) for 18 h. Prepared cells were then treated with TNTL.

### Migration assay

REC cells were seeded onto the apical side of transwell inserts (Corning, 8.0 μm pore size) with 150 μl serum-free medium. 750 μl serum-free medium containing indicated cytokines or collected cell supernatants were added to the receiving chamber and incubated for 6 h. Cells retained on the apical side of the insert membrane were removed, whereas cells at the basolateral side were fixed in 4% paraformaldehyde and stained with crystal violet. Images of migrated cells were captured under a light microscope.

### Barrier function assay

The function of endothelial cells was tested by Transepithelial/Transendothelial Electrical Resistance (TEER) and leakage of FITC-Dextran through the endothelial monolayer. RECs were seeded on the apical side of transwell inserts (Corning, 0.4 μm pore size) and grown until full confluence. The endothelial monolayer was incubated with culture supernatant of treated BMDMs for 48 h. TEER of the endothelial monolayer was tested by Ohm’s Law Method as described previously [22]. To test the endothelial monolayer leakage, 25 μg/ml FITC-Dextran (40 K, Sigma, USA) was added to the apical side of the monolayer followed by 24-h incubation. The concentration of FITC-Dextran at the upper and receiving chambers was determined by a luminescence spectrometer with excitation wavelength at 494 nm and emission wavelength at 518 nm. The percentage of FITC-Dextran leaked into the receiving chamber through the cell monolayer indicated the leakage after treatment [14].

### Quantitative real-time polymerase chain reaction (qRT-PCR)

Total RNA was extracted by Trizol (Takara, Japan) and cDNA was synthesized from total RNA with first strand synthesis kit (Takara, Japan). The mRNA expression of target genes was quantitatively measured with SYBR Green reagents (Takara, Japan) on the LC480 platform (Roche, USA). Targeted genes expression data were normalized by the expression level of β-actin. Primer sequence would be available upon request.

### Immunoblotting

Protein was extracted with RIPA buffer. 30 μg total protein was loaded onto SDS-PAGE and separated by electrophoresis. Separated proteins were then transferred to PVDF membrane followed by blocking with 5% BSA in TBST buffer (25 mM Tris-HCl, 137 mM NaCl, and 2.7 mM KCl, 0.05% Tween-20, pH 7.4 ± 0.2) for 2 h at room temperature. Membranes were then incubated with primary antibody of interests overnight at 4 °C and appropriate secondary antibody for 2 h at room temperature. The membrane was probed with ECL select substrate (GE Healthcare, Germany) on the Chemidoc chemiluminescent platform (Biorad, USA).

### Statistical analysis

For the animal study, the sample size was 6 in each group; for in vitro experiment, studies were performed in triplicate. Data was present in mean ± SD. For multiple group comparison, differences were measured with an ordinary two-way ANOVA with LSD multiple comparisons, while for comparison between two groups, differences were measured with student t-test. *p* < 0.05 were considered as statistically significant.

## Results

### High dose treatment of TNTL improved hyperglycemia in diabetic mice

**Fig. 1:**
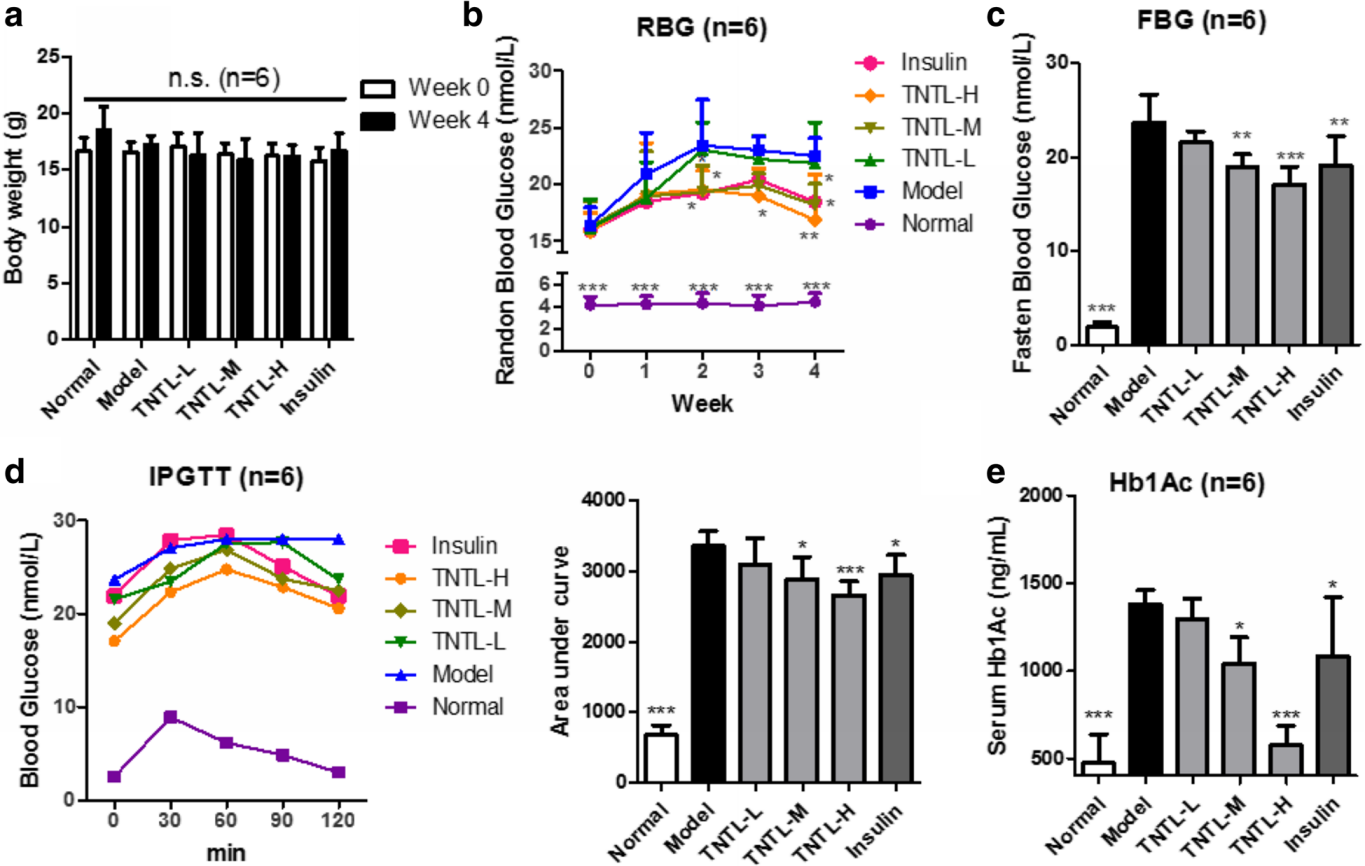
High doses of TNTL possessed hypoglycemic effects. **a**. showed that TNTL treatment did not significantly change the body weight of hyperglycemic mice; **b**. showed that TNTL dose-dependently suppressed the increase of random blood glucose (RBG) by week; **c**. showed that TNTL dose-dependently reduced fasting blood glucose (FBG) after 4-week treatment; **d**. showed that medium and high doses of TNTL improved the glucose tolerance in hyperglycemic mice; **e**. showed that TNTL at medium and high doses can significantly reduce serum HbA1c levels. *p < 0.05, **p < 0.01, ***p < 0.001 when compared with model group

Several previous studies have proven that TNTL improved insulin sensitivity and reduced blood glucose in type II diabetic models [23, 24]. To ensure the biological similarity of TNTL between batches, we firstly systemically evaluated the anti-hyperglycemic effect of TNTL on insulin-deficient type I diabetic model. Mice were continuously injected with a low dose of STZ (55 mg/kg, i.p.) for 5 days, and those with FBG within 15 to 20 mmol/L were treated with different doses of TNTL. It was found that oral TNTL treatment had minimal impact on the body weight of diabetic mice (Fig. 1a) but exhibited the dose-dependent manner in reducing the random blood glucose (RBG) of diabetic mice (Fig. 1b). While the lower dose of TNTL (0.9 g/kg, TNTL-L) showed no significant effect on the RBG of mice; medium and high doses (1.8 and 3.6 g/kg, TNTL-M and TNTL-H, respectively) could suppress the increase of blood glucose. 4-week treatment of TNTL at 1.8 and 3.6 g/kg significantly reduced the FBG, and this effect appeared to be compatible with insulin treatment (Fig. 1c). Further analysis of the glucose intolerance of mice showed that TNTL treatment at 1.8 and 3.6 g/kg significantly enhanced the tolerance of mice to glucose (Fig. 1d). Furthermore, TNTL dose-dependently reduced the plasma level of HbA1c, a marker used for risk estimates of diabetic complications (Fig. 1e). These data suggested that different batches of TNTL maintained consistent quality with similar biological activities, and this batch of herbal products was suitable for further experiment.

### TNTL suppressed the progression of DR independent to its anti-hyperglycemic effect

**Fig. 2:**
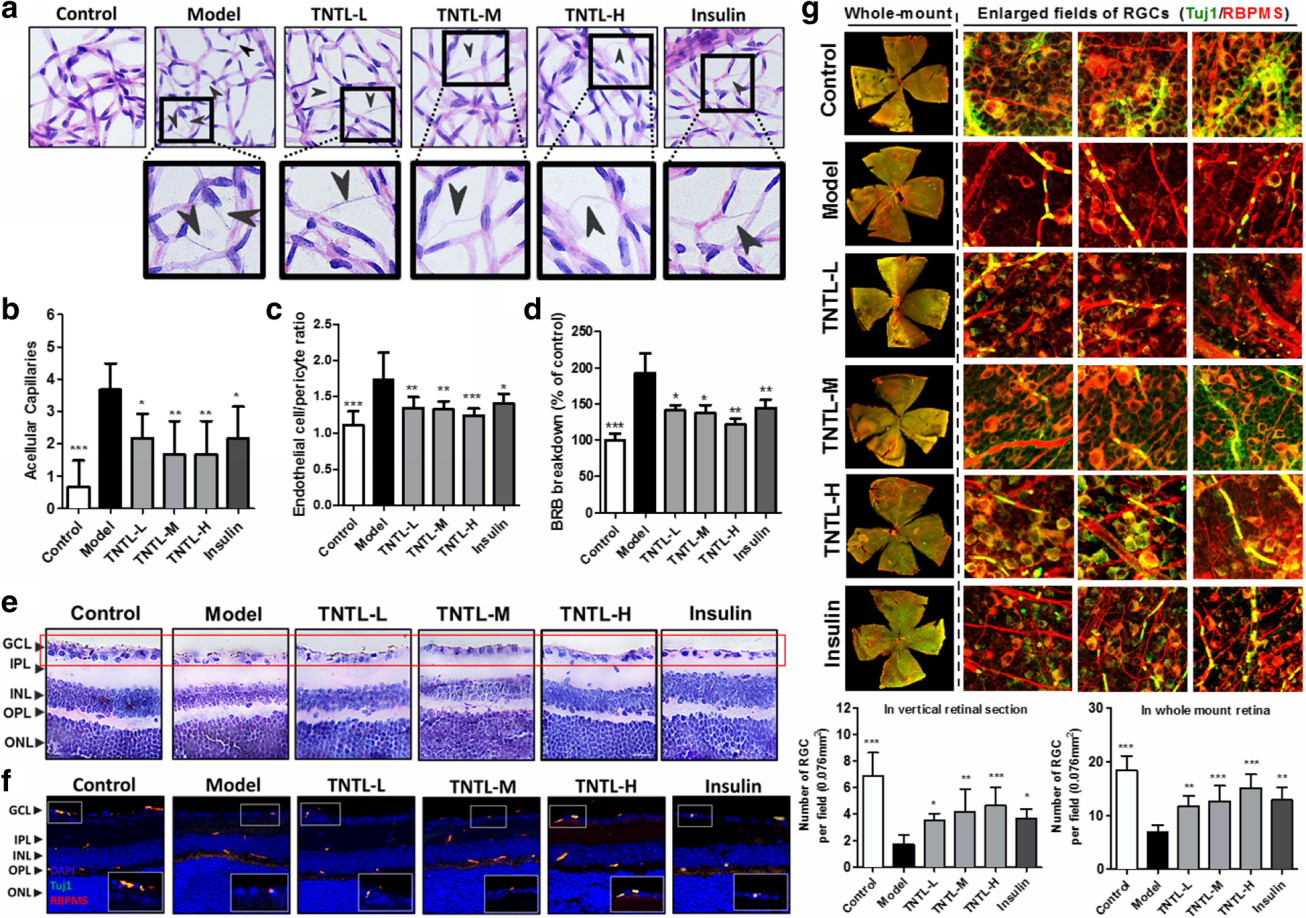
Non-hypoglycemic doses of TNTL improved retina condition in diabetic mice. **a**. showed the representative of retina vascular preparation from mice with various treatment; The block arrows showed the acellular capillaries; **b**. showed that low-to-high doses of TNTL could significantly reduce the number of acellular capillaries on the retina vascular; **c**. showed that low-to-high doses of TNTL could significantly reduce the ratio of endothelial cell/pericytes; **d**. showed that TNTL improved blood-retina barrier (BRB) integrity and reduced Evans blue dye leakage; **e**. showed that low-to-high doses of TNTL could restore the number of retinal ganglion cells. **f**. co-immunostaining of Tuj1 and RBPMS showed that retinal ganglion cells were recovered by TTNL at ganglion cell layer; **g**. showed that TNTL recovered the density of Tuj1/RBPMS-positive retina ganglion cell in the in whole mount retinas. *p < 0.05, **p < 0.01 when compared with model group

DR is the major microvascular complication in diabetic patients. Proper glycemic control in patients with diabetes mellitus correlates to the amelioration of DR incidence [25]. Retinal vasculature was prepared and imaged (Fig. 2a), and the result showed that TNTL treatment, even at a low dose (0.9 g/kg), was capable of reducing the endothelial dysfunctions during DR progression, as characterized by increased acellular capillaries (Fig. 2b) and the endothelial cell-to-pericyte ratio (Fig. 2c). To observe whether TNTL improved the retina condition in type I diabetic mice, blood-retina barrier leakage was tested. It was showed that TNTL could dose-dependently recover the blood-retina barrier and prevent the leakage of circulating Evan blue dye into the eyes. TNTL at a lower dose failed to control hyperglycemia in mice, but its effective action in preventing the leakage indicated that the effect of TNTL in retarding DR progression might be independent to glycemic control (Fig. 2d). Furthermore, as the vision loss during progressive DR is directly associated with retinal neuron degeneration [26], we measured the integrity of retinal ganglion cells in mice with or without TNTL treatment. It was shown that TNTL treatment could potently maintain the number of ganglion cells in the retina of diabetic mice, which further confirmed the inhibition of DR progression induced by TNTL treatment (Fig. 2e). Also, we used double staining of two markers, Tuj1 and RBPMS, in the vertically sectioned eyecup slides and whole-mounted retina. Tuj1 is a neuron marker and was used to identify retinal ganglion cells [27]. RBPMS was recently identified as a specific marker of retinal ganglion cells in rodents (mice, rats, rabbits, and Hartley guinea pigs) [28, 29] as well as in cats and monkeys [29, 30]. Specifically, RBPMS was recently used to recognize retinal ganglion cells in the samples from patients with diabetic retinopathy [31]. It was shown that the Tuj1- and RBPMS-positive cells were mostly overlapped and located at the ganglion cell layer, which was consistent with a previous report [16]. In hyperglycemic mice, Tuj1/RBPMS-positive cells were significantly reduced compared with normal mice. TNTL treatment could remarkably recover Tuj1/RBPMS-positive cells in the retina. Observation in both vertically sectioned eyecup slides and whole-mounted retina was consistent (Fig. 2f & g). These data suggested that TNTL may be able to prevent the incidence and progression of DR independent to glycemic control.

### Inhibition of DR progression by TNTL involved its regulation on retinal inflammation

**Fig. 3:**
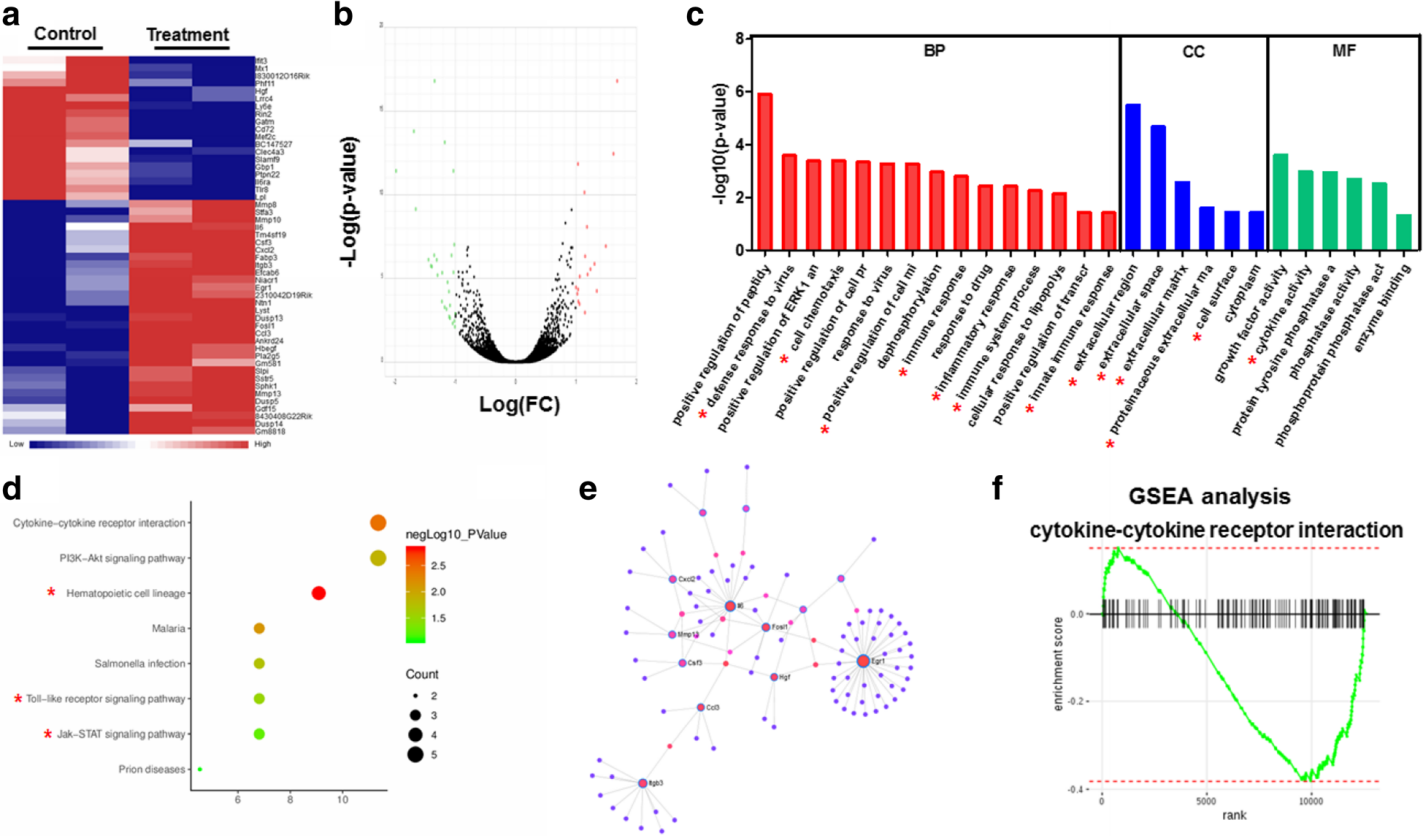
The inhibitory effect of TNTL on DR involves anti-inflammatory action. **a**. showed the heatmap of gene expression in the retina of hyperglycemia mice that were significantly changed after TNTL treatment; **b**. showed that volcano plot of gene expression profile. The green dots indicate genes with expression down-regulated by TNTL; red dots indicate genes with expression up-regulated by TNTL; while grey dots stand for genes without significant change in expression; **c**. showed the GO analysis on the involvement of main biological process (BP), cellular component (CC), and molecular function (MF) of common genes. The red asterisk indicated the GO items related to inflammation regulation; **d**. showed the KEGG pathway analysis on the common genes. **e**. showed the involvement of protein-protein interaction networks; **f**. showed GSEA analysis on the significantly changed gene-sets of cytokine-cytokine receptor interaction. It was revealed that TNTL treatment was negatively related to the identified gene sets

The initiation of DR, as well as its progression and end-stage vision loss, involves several physiopathological mechanisms such as biochemical, endocrine and hemodynamic anatomical alterations, which they interact with each other in a time sequence [32]. Among these changes, inflammation has been considered as a central role player in the initiation and development of DR in DM patients. Accumulating clinical evidence have proven the association between inflammation and clinical features of DR. To further understand the targeted molecules that involve in the regulation of retinal microenvironment by TNTL in hyperglycemic mice, we isolated retina from hyperglycemic mice treated with or without TNTL, and implemented transcriptomic analysis on the gene expression within retina tissues. It was showed that TNTL treatment could significantly induce a change of expression to a series of retina genes (Fig. 3a & b). Enrichment on the biological process (BP), cellular components (CC) and molecular functions (MF) related to expression change of the genes revealed that most of these genes play a common role in inflammation-related actions (Fig. 3c, highlighted with a red asterisk). Further analysis of TNTL-regulated signaling pathways on diabetic retina confirmed the involvement of inflammation-associated pathways (Fig. 3d, highlighted with a red asterisk). Also, by constructing the interaction network of proteins encoded by TNTL-altered retina genes, we observed that proteins associated with TNTL intervention played a key role as nodes in the construction of protein-protein interaction network related to inflammation (Fig. 3e). GSEA analysis further proved that TNTL treatment was prone to suppress inflammatory-related gene expression in the retina of hyperglycemic mice (Fig. 3f). These findings suggested that inhibition of DR by TNTL may involve its regulation on the pro-inflammatory retinal microenvironment.

### Macrophage suppression by TNTL prevented endothelial dysfunction in the retinal microenvironment

**Fig. 4:**
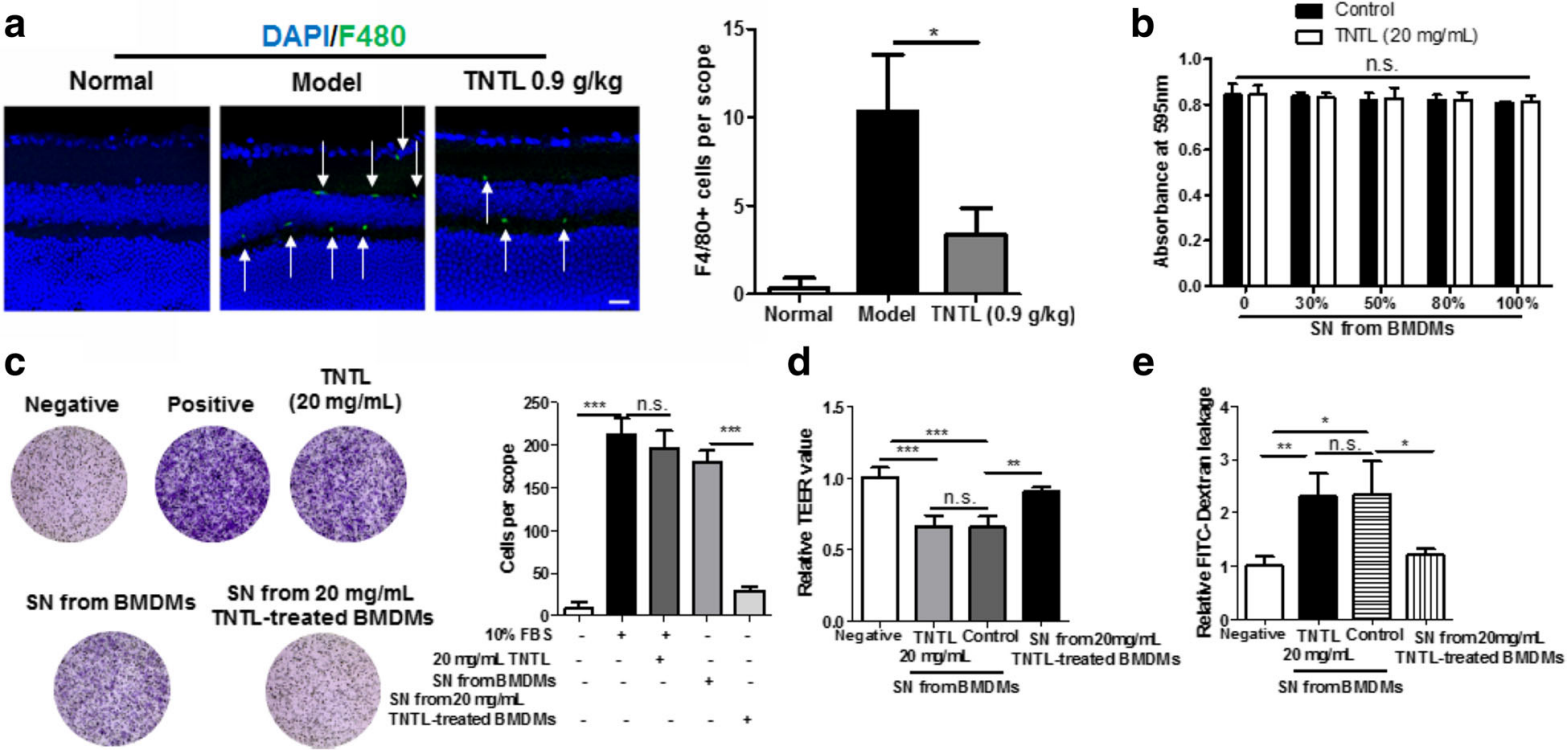
Suppression of pro-inflammatory macrophages by TNTL improved the endothelial condition in retina. **a**. showed that TNTL treatment could significantly reduce the infiltration and residence of F4/80+ macrophages; **b**. showed that supernatant (SN) from TNTL-treated pro-inflammatory BMDMs had minimal effect on the cell viability of retinal endothelial cells (RECs); **c**. showed that SN from TNTL-treated pro-inflammatory BMDMs failed in inducing RECs migration; **d**. showed that SN from TNTL-treated pro-inflammatory BMDMs induced loss of monolayer integrity formed by RECs; **e**. showed that SN from TNTL-treated pro-inflammatory BMDMs led to the leakage of FITC-Dextran through RECs monolayers. *p < 0.05, **p < 0.01, ***p < 0.001 when compared with model group

Inflammation in the retinal microenvironment was majorly contributed by the infiltration of macrophages and release of pro-inflammatory cytokines [33]. To examine whether TNTL suppressed inflammation in the retinal microenvironment, immunofluorescence staining of macrophage marker F4/80 was applied on the retinal section. It was found that in the retina of hyperglycemic mice, resident and infiltrated macrophages were accumulated at the apical and basolateral sides of the inner nuclear layer, and TNTL treatment significantly reduced the macrophages population in the retina, indicating that the inflammatory microenvironment of the retina in hyperglycemic mice was suppressed (Fig. 4a). To understand if suppression of macrophage by TNTL contributes to its improvement on the retinal endothelial condition, pro-inflammatory BMDMs induced by IFNγ (20 ng/mL) and LPS (100 pg/mL) was treated with TNTL (20 mg/mL in water as a vehicle). The supernatant (SN) collected from BMDMs treated with or without TNTL was used to challenge RECs. It was shown that RECs cultured with TNTL-treated BMDMs derived SN had no significant difference on cell viability compared with RECs cultured with vehicle-treated BMDMs derived SN (Fig. 4b) but showed a potent reduction in motility (Fig. 4c). Furthermore, RECs monolayer cultured with TNTL-treated BMDMs derived SN exhibited higher integrity and lower leakage of FITC-dextran (Fig. 4d & e). TNTL itself shown the minimal effect on the migration, permeability, and integrity of RECs (Fig. 4c-e). As increased migration and loss of monolayer integrity indicated the endothelial dysfunction in the diabetic retina, these data suggested that the suppression of pro-inflammatory macrophages by TNTL may be involved in the prevention of endothelial dysfunction and DR progression.

### Suppression of MIP1γ/CCR1 axis by TNTL contributed to the prevention of retinal endothelial dysfunction

**Fig. 5:**
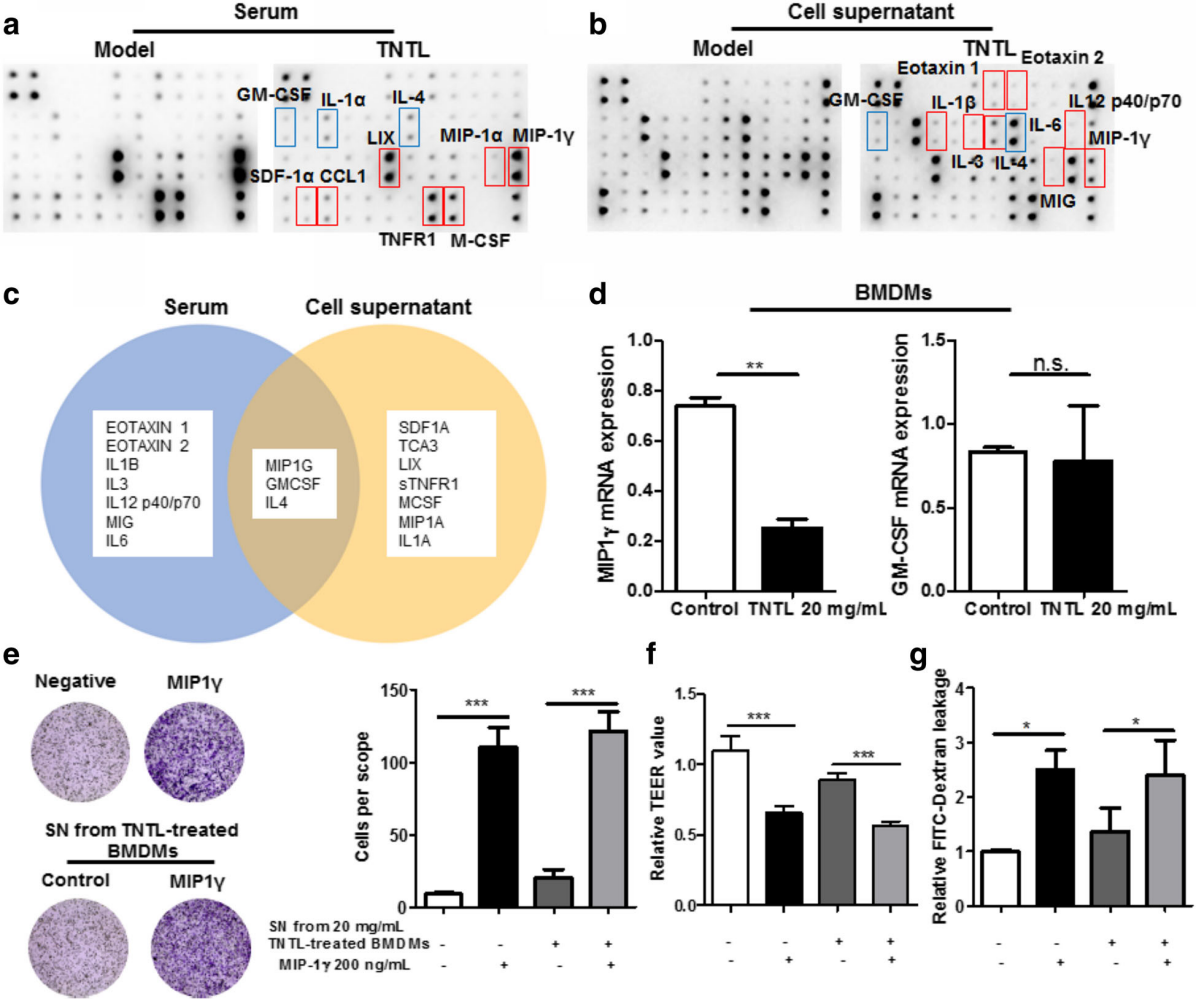
Suppression of TNTL on MIP1γ secretion from macrophages mediated its improvement on endothelial condition in retina. **a**. showed that antibody array on serums from hyperglycemic mice with or without TNTL treatment (0.9 g/kg); **b**. showed that antibody arrays on SN from BMDMs with or without TNTL treatment (20 mg/mL). The red squares highlighted cytokines suppressed by TNTL while the blue squares indicated cytokines provoked by TNTL; **c**. showed that IL4, GM-CSF, and MIP1γ were significantly regulated in both serum and SN; **d**. showed that expression of MIP1γ but not GM-CSF could be significantly suppressed in pro-inflammatory BMDMs by TNTL; **e**. showed that supplementation of MIP1γ in SN from TNTL-treated BMDMs recovered its ability in attracting RECs migration; **f**. showed that supplementation of MIP1γ in SN from TNTL-treated BMDMs recovered its ability in destroying RECs monolayer integrity; **g**. showed that supplementation of MIP1γ in SN from TNTL-treated BMDMs recovered its ability in inducing FITC leakage through RECs monolayer. *p < 0.05, **p < 0.01, ***p < 0.001 when compared with model group

To further explore the underlying anti-inflammatory mechanism of TNTL in preventing endothelial dysfunction in the retina, we conducted the antibody array on serum from mice treated with TNTL and SN collected from pro-inflammatory BMDMs treated with TNTL. The cytokines down-regulated by TNTL were highlighted in red while those up-regulated by TNTL were highlighted in blue (Fig. 5a & b). It was shown that TNTL consistently regulated three cytokines both in vitro and in vivo, namely MIP1γ, GM-CSF and IL4 (Fig. 5c). Since IL4 level was not consistently regulated in serum or SN by TNTL, expressions of MIP1γ and GM-CSF were further examined in TNTL-treated BMDMs. TNTL treatment showed significant suppression on MIP1γ but had minimal effect on GM-GSF (Fig. 5d). To further identify the critical target molecule, we supplemented the recombinant MIP1γ protein to the SN collected from TNTL-treated BMDMs. Using the modified SN to culture RECs, the inhibitory effect of TNTL on the REC migration was recovered by supplementation of recombinant MIP1γ protein (Fig. 5e). Consistently, loss of RECs monolayer integrity along with the increase of FITC-dextran leakage was observed in RECs cultured with MIP1γ-supplemented SN (Fig. 5f & g). These data suggested that suppression of MIP1γ release from macrophages by TNTL may be responsible for the prevention of endothelial dysfunction in the diabetic retina.

**Fig. 6:**
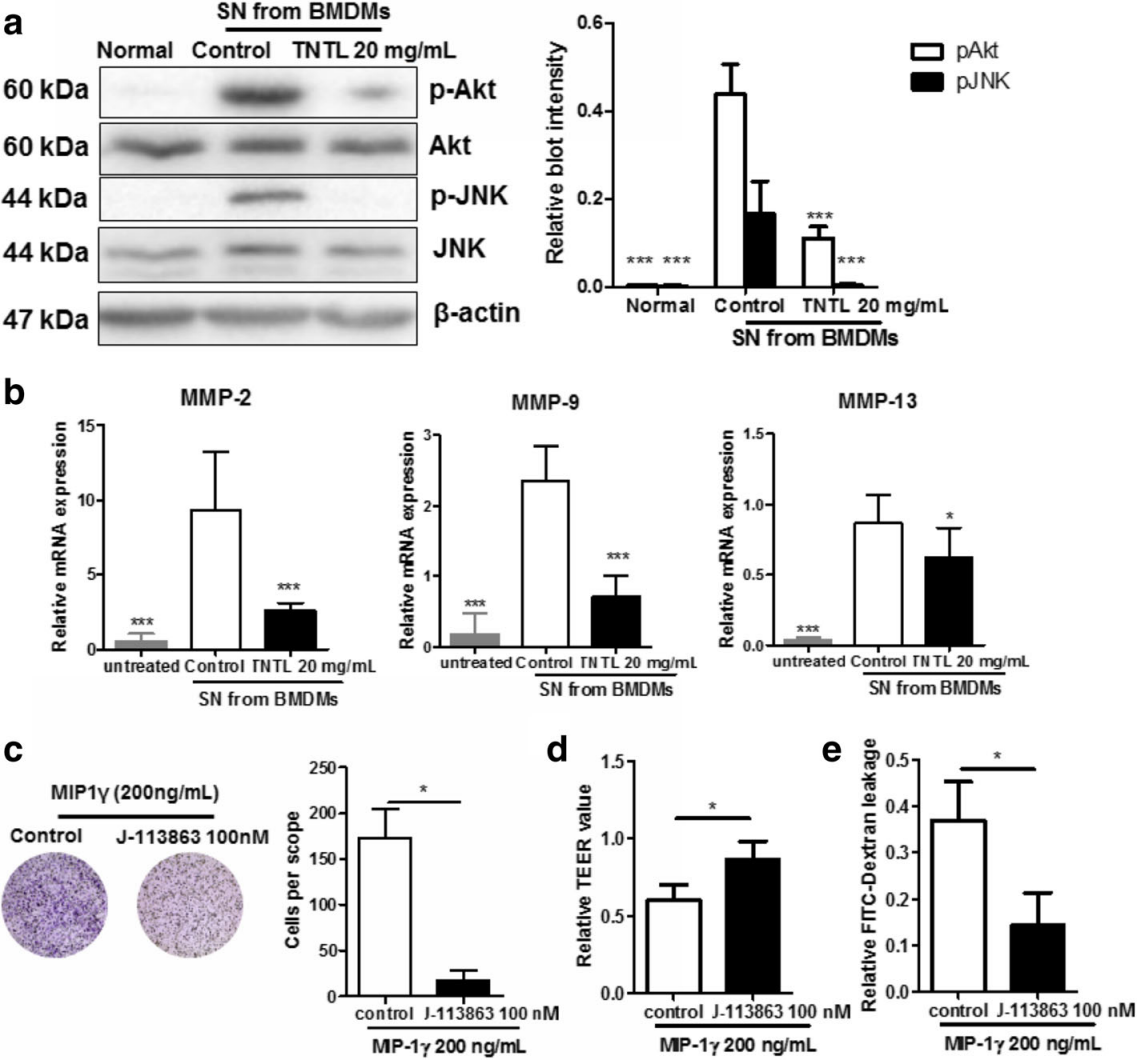
MIP1γ/CCR1 axis in retina endothelial cells contributed to the ameliorative effect of TNTL on endothelial dysfunction. **a**. showed that CCR1 downstream signalings in RECs cultured with SN from TNTL-treated BMDMs were blunted; **b**. showed that transcription of migration/invasion-associated genes was not activated in RECs cultured with SN from TNTL-treated BMDMs; **c**. showed that MIP1γ-induced RECs migration was blocked by the presence of CCR1 antagonist J-113863 (100 nM); **d**. showed that MIP1γ-induced loss of RECs monolayer integrity was blocked by the presence of CCR1 antagonist J-113863 (100 nM); **e**. showed MIP1γ-induced FITC-Dextran leakage through RECs monolayer was blocked by the presence of CCR1 antagonist J-113863 (100 nM). *p < 0.05, **p < 0.01, ***p < 0.001 when compared with model group

MIP1γ is a cytokine constitutively secreted by macrophages and myeloid cells [34]. MIP1γ and its receptor CCR1, has been widely reported for their ability in regulating cell motility [35]. To examine the involvement of MIP1γ/CCR1 pathway in the anti-inflammatory effect of TNTL, firstly, we blotted the related molecules within RECs cultured with SN from TNTL-treated BMDMs. It was showed that CCR1 mediated downstream pathways, such as JNK and PI3K/Akt, was less activated when BMDMs were pre-treated by TNTL (Fig. 6a). This claim was further confirmed by qRT-PCR assay, which showed reduced expression of migration/invasion-associated transcription products of MIP1γ/CCR1 pathway (Fig. 6b). Furthermore, migration of RECs provoked by MIP1γ was blocked by CCR1 antagonist J-113863 [36] (Fig. 6c), and loss of monolayer integrity and leakage of FITC-dextran by MIP1γ were reversed by J-113863 (Fig. 6d & e), Indicating that blocking MIP1γ/CCR1 pathway in endothelial cells may prevent the inflammatory macrophage-induced endothelial dysfunction. These data, combined with other above findings, suggest that inhibition of MIP1γ/CCR1 axis in retinal microenvironment by TNTL contributed to the inhibition of DR progression.

## Discussion

Insulin therapy remains to be an effective mean for glycemic control in diabetic patients. However, insulin therapy is deemed to be unsuccessful in well controlling the incidence of DR, conversely, acts as one of the contributors of DR [37]. Insulin use is an independent risk factor of DR progression in US adults with diabetes (OR, 3.23; 95% CI, 1.99–5.26) [38]. The previous study showed that the majority of type II diabetic patients using insulin injection suffered from DR (70%) compared to those without insulin treatment (39%) [39]. A meta-analysis on seven cohort studies detected a significant association between insulin usage and increased risk of DR despite existing heterogeneity of retrieved literature [40]. Residual endogenous insulin secretion is associated with both DR prevalence and its severity in Latinos with familial type II diabetes [41]. These previous findings suggest that glycemic control may not be the only critical factor to prevent incidence of DR. We found that TNTL at its high doses may possess anti-hyperglycemic effect, however, improvement on the retina condition by TNTL could be steadily achieved at its low dose as well, which indicated that the effect of TNTL on DR might be independent, at least partially, to its blood glucose-reducing action. Currently, ocular injection of anti-VEGF monoclonal antibody is the main non-surgical approach in treating late-stage DR, and intravitreal corticosteroids injection targeting inflammation has been approved by FDA as an alternative treatment to patients without significant response to anti-VEGF treatment [42]. The current protocol for a combination of anti-VEGF and anti-inflammatory agents in treating DR is not available despite several trials to have been conducted [43], probably due to the assumption that frequent vitreous injection may not be practical and do harm to the eye condition, however, an additional benefit of combination treatment is still possible to DR patients. Some pro-inflammatory factors, such as angiopoietin-2, proteinases, CCL2, were suggested to be potential targets [44]. In our study, we found that inflammatory suppression instead of controlling the systemic condition of diabetes might be involved in the effect of TNTL, which may particularly exert a beneficial effect on the normalization of the retina environment by targeting MIP1γ/CCR1 axis. This indicates TNTL as an orally administrated anti-inflammatory agent can benefit retina condition in diabetes, and offers a possibility in the practice of combination treatment of TNTL with intravitreal injection of anti-VEGF agents. Indeed, several clinical studies about the efficacy and safety of TNTL on diabetic patients have been undergoing or completed (clinicaltrial.gov registration No. NCT02161276 & NCT02174042). Findings from our studying combining the clinical observation on efficacy and safety of TNTL may further justify its potential as an adjuvant therapy to intravitreal injection of anti-VEGF in DR treatment.

Although in our study, we found that TNTL exhibited a protective effect against hyperglycemia-induced retinopathy at the dose which it did not reduce blood glucose, it cannot fully conclude that the two actions could be separated. This could occur due to the low sensitivity of the assays but may also be due to the complicated crosstalk of hyperglycemia and inflammation in the mechanism initiating retinopathy during diabetes. It was previously shown that hyperglycemia could significantly provoke inflammation via an oxidative mechanism [45]. However, anti-inflammatory agents have also been proven to reduce blood glucose in diabetes [46]. This suggests that it might be difficult to completely distinguish the effect of anti-hyperglycemia and anti-inflammation in diabetic treatment. Furthermore, whether TNTL could control inflammation systematically or specifically in retina remains unclear. Although from in vitro study we confirmed that possible inhibitory effect of TNTL on immune-endothelial cell interaction, the conclusion about the target inhibition on retinal inflammation by TNTL in vivo could be comprised without ruling out the possibility of TNTL in controlling systemic inflammation. Some further studies, for example, to determine whether revoking systemic inflammation could comprise the effect of TNTL, may provide more justifications. Also, identifying active components from TNTL that could pass blood-retina barriers may also help to understand its target specific action.

An opportunity for prevention and treatment of DR by blocking inflammation has been partially evident by clinical observations on the use of corticosteroids in the treatment of proliferative DR. It was observed that intravitreal injection of triamcinolone before laser panretinal photocoagulation could significantly reduce the neovascularization [47] and was associated with reduced risk of proliferative DR worsening [48]. In patients with non-high-risk proliferative DR, intravitreal injection of triamcinolone as an adjuvant therapy could delay the deterioration of visual acuity [49], but this effect could be insignificant in patients with high-risk proliferative DR [50]. This may suggest that the intravitreal corticosteroids treatment could be still controversial. Indeed, several observations also reported that the effect of intravitreal injection of triamcinolone to reduce the rates of progression of proliferative DR was not warranted [51, 52]. Also, the application of corticosteroids faces several shortcomings. Frequent injection is necessary. High incidence of cataract and ocular complications such as cataract formation, IOP, and glaucoma as well as systemic side effects including exacerbation of diabetes, may exclude its prophylactic use in DM patients without retinopathy, as well as therapeutic application in the early stage of DR [53]. Treatment with the nonsteroidal anti-inflammatory drug was proposed, but the clinical study showed that only high dose of aspirin (900 mg/day) could minimize the development of the early stage of DR [54], while systemic COX-2 inhibitor increased the risk of heart attacks and strokes in DR patients [55]. In our study, we observed that TNTL possessed significant anti-inflammatory effect in DR. TNTL is ann herbal formula that has been used for thousands of years without apparent side effects. The acute and chronic impacts of TNTL on rodents were recently tested, which suggested that TNTL has no profound toxicity to the animals (data not shown). The present observation supported that TNTL, with a mechanism targeting on MIP1γ release from retinal macrophages that can further suppress endothelial activation, can be an alternative treatment for DR, and a potential lead for the discovery of novel anti-inflammatory agents that are suitable for DR treatment.

We noticed that in addition to pathways-related to inflammation and immune system, TNTL might also target on PI3K/Akt pathway. It was previously found that regulation on the PI3K/Akt pathway may play an essential role in the treatment of diabetic retinopathy. Sheikpranbabu et al. suggested that inhibition of PI3K/Akt could significantly suppress vascular permeability by improving junction protein expression [56]; controversially, activating PI3K/Akt signaling in retinal neural cells protected against hyperlipidemia-induced oxidative stress and cell death [57]. More interestingly, several previous studies were suggesting that PI3K/Akt could serve as important signaling in the activation of retinal pigment cells during disease progression. Inhibition of PI3K/Akt by (−)-epigallocatechin gallate could significantly inhibit the migration and adhesion of retinal pigment cells [58]. Interestingly, a few studies also suggested that PI3K/Akt may be involved in the inflammation-related retinal pigment cell activation. A pro-inflammatory factor high mobility group B1 could induce secretion of angiogenic and fibrogenic factors in retinal pigment cells through PI3K/Akt activation [59]. High glucose may cause secretion of pro-inflammatory cytokines from retinal pigment cells, which could be related to PI3K/Akt activation [60]. These suggest that regulating PI3K/Akt alone or in combination with anti-inflammatory treatment may be a novel approach in DR management.

We found that macrophage-secreting MIP1γ may mediate the inhibitory effect of TNTL on endothelial dysfunction. The role of MIP1γ in the initiation and progression of DR has yet been understood, however, as a well-known small cytokine secreted by macrophages and myeloid cells, MIP1γ induced movement and infiltration of macrophages during inflammation [61]. Apart from its role as a macrophage attractant, MIP1γ was found to promote and maintain the differentiation and survival of osteoclasts in osteoclastogenesis [62, 63], which indicates its involvement in various human diseases. The primary receptor of MIP1γ on the cellular surface is CCR1, which was found to be expressed in different types of cells, including endothelial cells [64]. Binding of ligands to CCR1 activates the intracellular signaling and induces migration of endothelial cells and live capillaries without affecting its viability and proliferation [65, 66], which in turn leads to neovascularization and angiogenesis [64]. Thus, CCR1 activation in mediating endothelial cell migration without affecting its viability supported our observations that TNTL-treated SN lacking MIP1γ could influence RECs migration but had minimal effect on the cell survival compared with control SN with MIP1γ. Further observation showed that matrix metalloproteinases (MMPs) expression in RECs cultured with TNTL-treated BMDMs derived SN was reduced. Interestingly, it was previously found that both MIP1γ stimulation and CCR1 activation induce MMPs and promote cell migration and invasion [67, 68], and this effect of MIP1γ was blocked by the presence of CCR1 inhibitor as observed in our study, suggesting that MIP1γ/CCR1 axis play an essential role in mediating the activation of cell migration/invasion-associated signaling in endothelial cells. Compared with the application of CCR1 inhibitor in blocking DR progression with the possibility of side effects in animal and human, the strategy targeting MIP1γ to inhibit activation of CCR1-associated signaling in retinal endothelial cells may be potential to strengthen and diversify treatments against DR progression.

## Conclusion

**Fig. 7:**
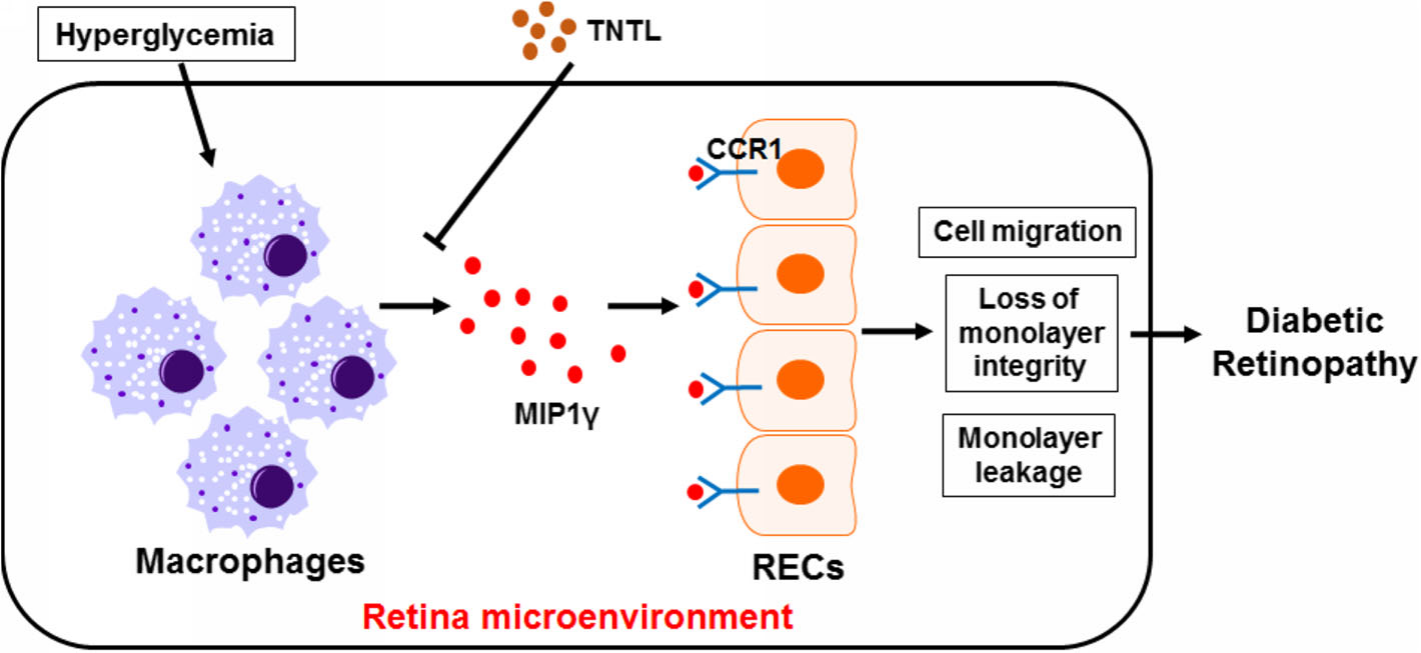
Proposed mechanism underlying the action of TNTL in treating DR

In conclusion, in this study, we explore the novel therapeutic target of diabetic retinopathy in the intervention of a botanical formula TNTL via transcriptomic analysis in combination with protein array. Consistent quality of TNTL extract was validated by its similar biological activity on hyperglycemia with that reported in previous studies. At its doses without significantly reducing blood glucose in hyperglycemic mice, TNTL was able to ameliorate the initiation and progression of DR, as evidenced by reduced BRB leakage, retinal neovascularization and restored retinal ganglion population. We performed transcriptomic analysis on the gene expression in the retina of hyperglycemic mice with or without TNTL treatment and found that TNTL may inhibit inflammation in the retinal microenvironment. The anti-inflammatory effect of TNTL was validated by experimental observations of reduced infiltration of pro-inflammatory macrophages in the retina. Suppression of inflammation in macrophages reduced the secretion of factors that promoted migration and loss of endothelial cells integrity. Also, antibody array analysis suggested that MIP1γ, whose secretion by macrophage was inhibited by TNTL, may be responsible for the therapeutic effects. Supplementation of MIP1γ neutralized the effect of TNTL on endothelial dysfunction. CCR1-associated pathway in endothelial cells might be responsible for the inhibitory effect of TNTL on MIP1γ-induced disorders in endotheliocytes (Fig. 7). Our study suggested that TNTL may improve DR by targeting MIP1γ/CCR1 axis to reduce inflammation in the retinal microenvironment.

## Data Availability

The datasets used and/or analyzed during the current study are available from the corresponding author on reasonable request.

## Abbreviations

BMDMs: Bone marrow-derived macrophages
BP: Biological process
BRB: Blood-retina barrier
CC: Cellular components
CCR1: C-C chemokine receptor type 1
CI: Confidence interval
COX-2: Cyclooxygenase-2
CRP: C-reactive protein
DM: Diabetes mellitus
DME: Diabetic macular edema
DR: Diabetic retinopathy
ECL: Enhanced Chemiluminescence
FBG: Fasting blood glucose
GMP: Good manufacturing practice
GO: Gene ontology
GSEA: Gene set enrichment analysis
HbA1c: Hemoglobin A1c
HIF-1α: Hypoxia-inducible factor-1α
IFNγ: Interferon-γ
IL1β: Interleukin-1β
IL4: Interleukin-4
IL6: Interleukin-6
IL8: Interleukin-8
IOP: Intraocular pressure
JNK: c-Jun N-terminal kinase
KEGG: Kyoto Encyclopedia of Genes and Genomes
LPS: Lipopolysaccharide
MCP-1: Monocyte chemoattractant protein-1
M-CSF: Granulocyte-macrophage colony-stimulating factor
M-CSF: Macrophage-colony stimulating factor
MF: Molecular functions
MIP1γ: Macrophage inflammatory protein-1 γ
OR: Odds ratio
PI3K: Phosphoinositide 3-kinase
RBG: Random blood glucose
RECs: Retinal Endothelial Cells
RIPA: Radioimmunoprecipitation assay
SDS-PAGE: Sodium dodecyl sulphate-polyacrylamide gel electrophoresis
sIL2R: Soluble interleukin-2 receptor
SN: Supernatant
STZ: Streptozotocin
TBST: Tris-buffered saline-Tween 20
TEER: Transepithelial/Transendothelial Electrical Resistance
TNFα: Tumor necrosis factor-α
TNTL: Tang-Ning-Tong-Luo
VEGF: Vascular endothelial growth factor
